# Sparse Coding Using the Locally Competitive Algorithm on the TrueNorth Neurosynaptic System

**DOI:** 10.3389/fnins.2019.00754

**Published:** 2019-07-23

**Authors:** Kaitlin L. Fair, Daniel R. Mendat, Andreas G. Andreou, Christopher J. Rozell, Justin Romberg, David V. Anderson

**Affiliations:** ^1^School of Electrical and Computer Engineering, Georgia Institute of Technology, Atlanta, GA, United States; ^2^Department of Electrical and Computer Engineering, The Johns Hopkins University, Baltimore, MD, United States

**Keywords:** sparsity, sparse-approximation, sparse-code, brain-inspired, TrueNorth, spiking-neurons

## Abstract

The Locally Competitive Algorithm (LCA) is a biologically plausible computational architecture for sparse coding, where a signal is represented as a linear combination of elements from an over-complete dictionary. In this paper we map the LCA algorithm on the brain-inspired, IBM TrueNorth Neurosynaptic System. We discuss data structures and representation as well as the architecture of functional processing units that perform non-linear threshold, vector-matrix multiplication. We also present the design of the micro-architectural units that facilitate the implementation of dynamical based iterative algorithms. Experimental results with the LCA algorithm using the limited precision, fixed-point arithmetic on TrueNorth compare favorably with results using floating-point computations on a general purpose computer. The scaling of the LCA algorithm within the constraints of the TrueNorth is also discussed.

## 1. Introduction

Physiological evidence exists of sparse coding being employed by biological systems to achieve processing efficiency (Olshausen and Field, 2004). In sparse coding, redundancy in the environment is leveraged to produce efficient representations; therefore, to process a given stimuli, the number of firing neurons are minimized (Field, 1994; Olshausen and Field, 1996; Olshausen, 2003). Sparse codes are computed by solving for the sparse approximation of a signal. The sparse approximation is represented by the weights of each element from an over-complete dictionary, that can be employed to reconstruct the original signal via a linear combination of the elements. The dimensionality of the data is not reduced; however, the number of dictionary elements with non-zero weights are few relative to the dictionary size and hence a sparse representation. The Locally Competitive Algorithm (LCA) (Rozell et al., 2008) is a biologically plausible algorithm that solves the sparse approximation problem. Convergence of the algorithm to the correct solution is theoretically proven and guaranteed (Balavoine et al., 2012, 2013a,b; Shapero et al., 2014). In more general terms the LCA algorithm is a non-linear dynamical system, that computes a sparse approximation of a signal iterating in time until the desired solution is stable. Sparse approximation algorithms such as the LCA have applications in a number of signal enhancement and reconstruction applications, especially in image processing (Elad et al., 2010; Zibulevsky and Elad, 2010; Yang et al., 2013).

Using the LCA algorithm as a proof of concept, this work establishes a meaningful computational framework for implementing recurrent network architectures on the low-precision, neuromorphic IBM TrueNorth Neurosynaptic System (Merolla et al., 2014). A typical image patch, about 8 × 8 pixels, and a dictionary of 100 LCA nodes, uses only 113 of the 4096 cores available on the TrueNorth. Much larger dictionaries can also be programmed or the mapped algorithm can be implemented such that multiple image patches are processed in parallel. Results are presented in section 6. Previously, a recurrent neural network has been mapped to physically simple but highly-non-linear, analog circuit models of neurons and synapses (Pineda and Andreou, 1995). This problem was approached using a decompression algorithm for fractal block codes. Parameters in the non-linear system were chosen in a way that there was only one stable, correct solution, much like the LCA, but computed with only 16 neurons on a purely analog CMOS chip. Implementations of specifically the LCA as a recurrent network using integrate and fire neurons have been demonstrated on field-programmable analog arrays (FPAAs) (Shapero et al., 2012, 2013). These designs use small, proof-of-concept dictionaries, up to 18 LCA nodes. A theoretical scaling analysis of the LCA on the FPAA architecture could consume less power than the currently-implemented architecture for the same dictionary size (see section 7). However, no FPAA design has been realized to a scale comparable to that implemented on the TrueNorth system. More recent works (Olshausen and Rozell, 2017; Sheridan et al., 2017) describe memristor crossbar-based solutions for implementing the LCA. Even though thus far the latter work has applied to small image patches (4 × 4 pixels, 32 LCA nodes) this new avenue of research has the potential to eventually scale to larger dictionaries such as those implemented in this work. In the latter paper (Sheridan et al., 2017) the original recursive LCA algorithm was re-written in terms of two feedforward computations; a mathematical transformation that facilitates the hardware implementation on neuromorphic hardware.

In summary, the main contribution of this paper is a highly-scalable mapping of the neurally-inspired LCA algorithm to solve for the sparse code of a signal within the architectural framework of a crossbar-based, non-von Neumann biologically-inspired architecture, the IBM TrueNorth neurosynaptic system. The implementation described in this paper moves past proof-of-concept implementations and offers a highly scalable, sparse approximation solver for low-power, signal processing applications. The differences between a conventional computer architecture and the TrueNorth necessitate a novel design methodology to map the algorithmic structures of the LCA onto the TrueNorth system. The mapping of the LCA to the TrueNorth architecture in this work serves as a blueprint for further exploration of general techniques to map basic iterative linear algebra and dynamical systems based algorithms within the constraints of emerging computational architectures. Addressing the challenges of implementing basic iterative linear algebra in neuromorphic hardware such as the TrueNorth, which is available for experimentation and supported by software programming environments (Amir et al., 2013), lays the foundation for further exploration of computational architectures inspired by the nervous system for a wide range of applications in sensory processing and cognitive computing. These non-von Neumann combined hardware/software architectures are vital to improving computing capabilities as conventional CPU architectural improvements are plateauing. Programming in these new paradigms is not trivial, therefore developing tools for performing common computations using these hardware architectures provides building blocks to the community for further utilizing these new neuromorphic devices in other applications. This work advances computational sciences and is a step toward the engineering of truly cognitive machines (Cauwenberghs, 2013; Boahen, 2016) in the era “beyond Moore” (Bahar et al., 2007; Cassidy et al., 2013a; Cavin, 2015).

Section 2 presents the discrete time approximation of the LCA dynamics with variables scaled to account for the limited precision of the hardware. Section 3 outlines the TrueNorth neurosynaptic system architecture and section 4 addresses programming challenges with data representation. In section 5 we discuss three functional processing units that enable an efficient implementation of an iterative algorithm, i.e., dynamical data structures and locality of reference. These processing units include fixed-point integer vector-matrix multiplication, a non-linear threshold function, and dynamic memory. Results and discussion follow in section 6, 7, respectively.

## 2. Discrete Time Representation of LCA Dynamics

The LCA computes the sparse approximation, or sparse code, of a signal relative to a given over-complete dictionary. Each LCA neuron or node represents an element from the dictionary Φ combined to reconstruct a signal **y**(*t*) via its sparse approximation **a**(*t*): $\hat{\text{y}}\left(t\right)=\Phi \text{a}\left(t\right)$. The internal state of each node is contained in the vector **u**(*t*). The sparse approximation **a**(*t*) is computed by implementing a soft threshold on each node's internal state **u**(*t*), described in detail in Equation (2). The *m*th node's state changes according to the dynamics

(1) $$\begin{array}{l} {\dot{u}}_{m}\left(t\right)=\frac{1}{\tau }\left(b_{m}\left(t\right)-u_{m}\left(t\right)-\underset{m\neq k}{\sum }G_{m,k}a_{k}\left(t\right)\right). \end{array}$$

The value τ is a positive, system-determined time constant that controls how quickly the system converges to the sparse approximation. The lateral inhibition is performed by *G*_*m, k*_ on each node, calculated by taking the inner product of each node with all other nodes *G*_*m, k*_ = 〈Φ_*m*_, Φ_*k*_〉 where *m* ≠ *k*, such that a node does not inhibit itself in the computation. A concise representation of this inhibition is to calculate Φ^*T*^Φ and set diagonal values to zero, resulting in the inhibition matrix **G**. Larger values within this matrix signify more closely related nodes. Active nodes suppress nodes based on the values found in *G* to reduce redundancy in the sparse approximation of a signal. The initial projection of an LCA node *b*_*m*_(*t*) is calculated by *b*_*m*_(*t*) = 〈Φ_*m*_, **y**(*t*)〉. Due to the competitive nature of the node dynamics, **y**(*t*) can change with time and the LCA can converge to a new sparse approximation **a**(*t*), resulting also in a dynamic initial projection **b**(*t*).

To determine whether a node is active, meaning it contributes to the sparse approximation of the signal (Rozell et al., 2008), a soft threshold function is used. If the state of a node calculated by Equation (1) reaches or exceeds a threshold λ, the node becomes active and contributes to the sparse approximation while suppressing other similar node activity. The threshold λ serves as the tradeoff between sparsity and reconstruction accuracy. The soft threshold of the *n*th node is computed using

(2) $$\begin{array}{l} a_{k}(t)=T_{\lambda }(u_{k}(t))=\{\begin{array}{cc} \frac{u_{k}(t)}{g_{k}}-\text{sign}(u_{k}(t))\frac{\lambda }{g_{k}} & \text{if}|u_{k}(t)|\geq \lambda \\ 0 & \text{if}|u_{k}(t)|<\lambda . \end{array} \end{array}$$

The term *g*_*k*_ consists of the *n* diagonal values of Φ^*T*^Φ and is used for nodes with non-uniform norms. For a dictionary with unit norm nodes, *g*_*k*_ = 1 and the term can be disregarded in Equation (2).

To implement the LCA in a discrete time-step, limited precision hardware architecture such as the TrueNorth, we discretize the LCA algorithm by simple time-domain sampling

(3) $$\begin{array}{l} \text{u}\left[n+1\right]=\text{u}\left[n\right]+\Delta \text{u}\left[n\right], \end{array}$$

(4) $$\begin{array}{l} \Delta \text{u}\left[n\right]=\frac{1}{\tau }\left(\text{b}\left[n\right]-\text{u}\left[n\right]-\text{G}\text{a}\left[n\right]\right),\text{and} \end{array}$$

(5) $$\begin{array}{l} a_{k}[n]=T_{\lambda }(u_{k}[n])=\{\begin{array}{cc} \frac{u_{k}[n]}{g_{k}}-\text{sign}(u_{k}[n])\frac{\lambda }{g_{k}} & \text{if}|u_{k}[n]|\;\geq \lambda \\ 0 & \text{if}|u_{k}[n]|\;<\lambda . \end{array} \end{array}$$

All variables in Equations (3) to (5) represent the discrete-time values of the continuous-time variables in Equations (1) to (2). The value τ determines the convergence rate of the signal's sparse approximation. For the continuous dynamics system, τ ≈ 100 gives good convergence. To avoid the need for normalization by division on TrueNorth, we scale our system by τ^2^ and re-write equations as shown in Equations (6) to (8) such that all values are greater than or equal to one and can be accurately represented within a limited precision architecture. This scaling does not impact the rate of convergence, it only increases values in the system by a factor of τ^2^:

(6) $$\begin{array}{l} \tau^{2}\text{u}\left[n+1\right]=\tau^{2}\text{u}\left[n\right]+\tau^{2}\Delta \text{u}\left[n\right], \end{array}$$

(7) $$\begin{array}{l} \tau^{2}\Delta \text{u}\left[n\right]=\tau \text{b}\left[n\right]-\tau \text{u}\left[n\right]-\text{G}\tau \text{a}\left[n\right],\text{and} \end{array}$$

(8) $$\begin{array}{l} \tau a_{k}[n]=T_{\tau \lambda }(\tau u_{k}[n])=\{\begin{array}{cc} \frac{\tau u_{k}[n]}{g_{k}}-\text{sign}(u_{k}[n])\frac{\tau \lambda }{g_{k}} & \text{if}|\tau u_{k}[n]|\geq \tau \lambda \\ 0 & \text{if}|\tau u_{k}[n]|<\tau \lambda . \end{array} \end{array}$$

To implement these dynamics on the TrueNorth, the input to the system is the original signal **y**[*n*] and the output is the scaled sparse approximation τ**a**[*n*] in Equation (8). The discrete sparse approximation **a**[*n*] of the original signal is determined by taking the output of the system and dividing by τ, on chip using neuron thresholds or off chip manually. The original signal can be reconstructed using $\hat{\text{y}}\left[n\right]=\Phi \text{a}\left[n\right]$ on or off the TrueNorth.

## 3. TrueNorth Architecture Overview

The concept of neuromorphic systems (Mead, 1990) was introduced over two decades ago, defining such a system as one that is based on the organizing principles of the nervous system. The TrueNorth is a chip multi processor (Merolla et al., 2014) with a tightly coupled processor/memory architecture, that results in energy efficient neurocomputing and is a significant milestone to over 30 years of neuromorphic engineering (Cassidy et al., 2013a). The spiking neurons of the TrueNorth are representative of how the human brain efficiently represents and processes information (Cassidy et al., 2014). The TrueNorth architecture comprises 4096 cores each core with 65K of local memory (6T SRAM) or synapses and 256 arithmetic logic units, *neurons*, that operate on a *unary* number representation and compute by counting up to a maximum of 19 bits. The cores are event-driven using custom asynchronous and synchronous logic, and are globally connected through an asynchronous packet switched mesh network on chip (NOC). The chip development board includes a Zynq Xilinx FPGA that does the housekeeping and provides support for standard communication support through an Ethernet UDP interface. The asynchronous Addressed Event Representation (AER) in the NOC is also exposed to the user for connection to AER based peripherals through a packet with bundled data full duplex interface. The unary data values represented on the system buses can take on a wide variety of spatial and temporal encoding schemes. Pulse density coding (the number of events *N*_*e*_ represents a number N), thermometer coding, time-slot encoding, and stochastic encoding are examples. Additional low level interfaces are available for communicating directly with the TrueNorth to aid programming and parameter setting. A hierarchical, compositional programming language, *Corelet*, is available to aid the development of TrueNorth applications (Amir et al., 2013). IBM provides support and a development system as well as “Compass” a scalable simulator (Preissl et al., 2012). The software environment runs under standard Linux installations (Red Hat, CentOS, and Ubuntu) and has standard interfaces to Matlab and to Caffe (later switched to Eedn—energy—efficient deep neuromorphic networks) that is employed to train deep neural network models. The TrueNorth architecture can be interfaced using native AER to a number of bio-inspired sensory devices developed over many years of neuromorphic engineering (silicon retinas and silicon cochleas). In addition, the architecture is well suited for implementing deep neural networks with many applications in computer vision, speech recognition, and language processing.

Each TrueNorth core consists of 256 axons, 256 neurons, and 256 × 256 synaptic connections between the two (Cassidy et al., 2014; Merolla et al., 2014). An axon type is assigned to each axon, 23 programmable parameters and one axon destination are defined for each neuron, and synaptic connections between axons and neurons are chosen. Each axon *i* is assigned one type *G*_*i*_ ∈ {0, 1, 2, 3}. Each neuron *j* can assign one integer between –255 and +255 to the four axon types, labeled $s_{j}^{G_{i}}$, and can be thought of as synaptic weights if the synaptic connection between the axon and neuron is active. These four assignments can vary for different neurons. Synaptic connections *w*_*i, j*_ are binary.

Once the chip has been programmed, information is processed through the system using a unary data representation consisting of spikes that are routed between cores. Each neuron output can target only one axon input within any core in the system. If a neuron's membrane potential *V*_*j*_ reaches its threshold α_*j*_, it sends a spike to the appropriate axon *A*_*i*_. For timestep *n*, if an axon has received a spike, *A*_*i*_[*n*] = 1; otherwise, *A*_*i*_[*n*] = 0. The cores update neuron states every 1 ms for real-time operation. This clock can be sped up, but doing so can cause spike delivery to be delayed such that simulation results of the system are no longer equivalent to real hardware operation (Cassidy et al., 2013b; Merolla et al., 2014). A simplified version of TrueNorth neuron dynamics for neuron *j* at timestep *n* is shown in Equation (9):

(9) $$\begin{array}{l} V_{j}\left[n\right]=V_{j}\left[n-1\right]+\overset{255}{\underset{i=0}{\sum }}A_{i}\left[n\right]\times w_{i,j}\times s_{j}^{G_{i}}. \end{array}$$

**Figure 1:**
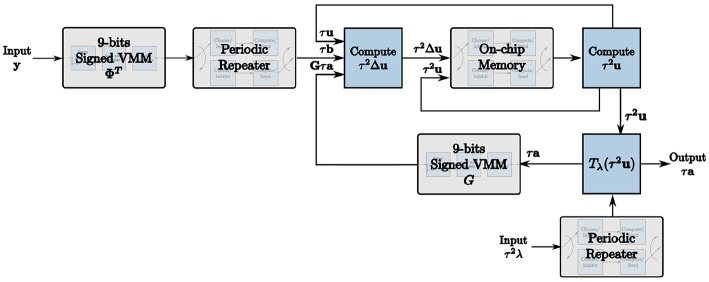
The corelet used to implement the LCA on the TrueNorth using our novel design methodology.

We exploit these programmable properties of TrueNorth neurons to map the LCA node dynamics to the hardware architecture, resulting in the LCA corelet in Figure 1. The computational units required to build this corelet are described in detail in section 4.

## 4. Challenges in Mapping LCA on TrueNorth

### 4.1. Encoding Increased Precision Values

In recurrent algorithms, values are constantly evolving. To map dynamic values onto the TrueNorth where neuron parameters are static, we must encode data to achieve increased precision. In the context of LCA, values **u**, Δ**u**, and **a** are constantly evolving until the LCA system converges. This prevents the use of programmable synaptic weights offered by the TrueNorth directly. For instance, suppose we are performing the summation in Equation (7). Ideally, for the LCA node *m* we would connect three TrueNorth axons to the same TrueNorth neuron, one axon to represent τ*u*_*m*_[*n*], one to represent τ*b*_*m*_[*n*], and the last to represent the *m*th value of τ**Ga**[*n*]. We would set the synaptic weights of the axons to be −τ*u*_*m*_[*n*], τ*b*_*m*_[*n*], and the *m*th value of −τ**Ga**[*n*], respectively. The output spikes of the neuron would therefore represent the solution $\tau^{2}\Delta u_{m}\left[n\right]$. However, all terms in the summation evolve as the input changes and the system converges, whereas the synaptic weights cannot be changed once the chip has been programmed.

**Figure 2:**
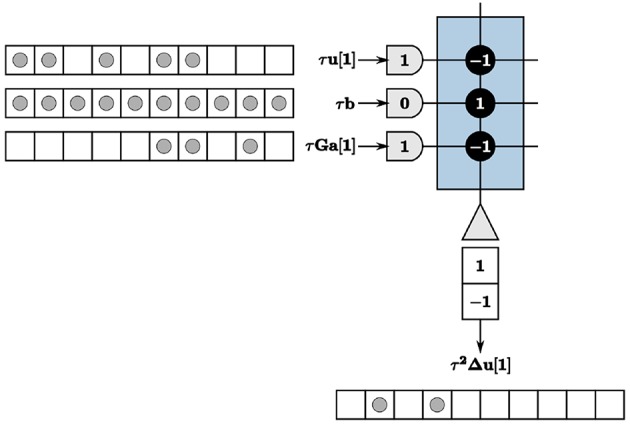
The summation computation is accurate for every iteration so long as values do not exceed the window size, *w* = 10.

We therefore encode values using a time window for each LCA iteration. The value of an LCA variable at each iteration is determined by counting the number of spikes within the given time window. Spikes can occur anywhere within the window to contribute to the resultant value. We use a window of *w* ticks for each LCA iteration, where *w* is greater than or equal to the largest value you would expect to see in a system. We show our encoding techniques in Figure 2 with *w* = 10, axons denoted by half-circles, neurons denoted by triangles, synaptic connections between the two denoted by solid black circles with weights overlaid for clarity, incoming and outgoing spikes represented as solid gray circles, and the time window represented by unfilled white blocks to make up a row of 10 blocks, each block being one TrueNorth tick. For this example, τ*u*_*m*_[1] = 5, and the *m*th value of τ**Ga**[1] = 3. We assume a constant input and therefore τ*b*_*m*_ = 10 for all time *n*. Synaptic weights for τ*u*_*m*_[*n*] and the *m*th value of τ**Ga**[*n*] are assigned as negative one and for τ*b*_*m*_ as positive one to accurately compute the update $\tau^{2}\Delta u_{m}\left[n\right]=2$ for this example.

### 4.2. Representing Signed Values

**Figure 3:**
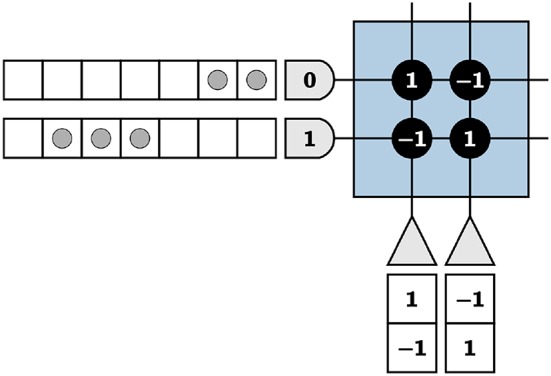
The left neuron is the positive representation of a variable and the right, the negative of the same variable.

TrueNorth neurons cannot emit negative spikes, and while biologically plausible in the context of biological systems' neurons, this requires TrueNorth neurons to be repeated so that for each value, one neuron output represents the standard positive spikes while an additional neuron is assigned to represent negative numbers. For instance, if the true value of an element is positive, we expect output spikes from the neuron that represents the positive values of that element. If the true value of the element is negative, we instead expect output spikes from the neuron that represents negative values. To successfully implement this technique, the positive representation neuron is repeated with the signs of the synaptic weights reversed, resulting in a negative representation of the value shown in Figure 3.

For now, to motivate our methods used to represent positive and negative values, we incorrectly assume TrueNorth neuron potentials *V*_*j*_ are reset by

(10) $$\begin{array}{l} V_{j}[n+1]=\{\begin{array}{cc} \text{spike},V_{j}[n]-\alpha & \text{if}\;V_{j}[n]\geq \alpha \\ V_{j}[n]+\beta & \text{if}\;V_{j}[n]\leq -\beta \\ V_{j}[n] & \text{if}-\beta <V_{j}[n]<\alpha , \end{array} \end{array}$$

where α is the positive threshold and β is the negative threshold for the neuron. It is important to note that upon reaching the negative threshold, a neuron potential resets without the neuron emitting spikes. Using Figure 3 as an example, we choose α, β = 1 and send two input spikes into the first axon and three input spikes into the second. The input spikes represent an input of [2 3], calculated by counting the number of spikes in a given time window. For this example, we use a window of seven TrueNorth ticks. We calculate output values in the same manner.

With neuron dynamics from Equation (10), the first neuron representing the positive value will emit two spikes at ticks two and three (due to the one tick delay on TrueNorth), the second neuron representing the negative value will emit three spikes at ticks five, six, and seven, and both neurons will have neuron potentials of zero at the end of the time window. In Figure 3, the vector [2 3] is encoded by input spikes and multiplied by [1 –1]^*T*^, shown by the synaptic weights of the first neuron. The total output of the negative representation neuron in *w* ticks is subtracted from the total output of the positive representation neuron in the same *w* ticks to find the final true value, for this example being 2 − 3 = 1.

This programming technique is promising; however, the actual TrueNorth neuron resets have asymmetric thresholds and pose a complication. Rather than membrane potentials resetting upon the potential being less than or equal to the negative threshold, they only reset once they drop below the negative threshold, while the positive thresholds reset once they meet the threshold, as we show in Equation (10). The thresholds in the TrueNorth chip actually have the following neuronal behavior:

(11) $$\begin{array}{l} V_{j}[n+1]=\{\begin{array}{cc} \text{spike, }V_{j}[n]-\alpha & \text{if }V_{j}[n]\geq \alpha \\ V_{j}[n]+\beta & \text{if }V_{j}[n]<-\beta \\ V_{j}[n] & \text{if }-\beta \leq V_{j}[n]<\alpha . \end{array} \end{array}$$

As a result, the first neuron emits two spikes at ticks two and three as before, but the second neuron will emit only two spikes at ticks six and seven. This produces an incorrect final value of 2 − 2 = 0. The final neuron potentials are –1 and zero for the first and second neurons at the end of the time window respectively, meaning that if these same neurons are used for computations in the next time window, the subsequent calculations will be incorrect due to the asymmetric thresholds not producing identical neuron states with opposite signs for positive and negative representations.

For values to take on different polarities over time on the TrueNorth chip, the positive and negative thresholds must be symmetric, essentially resetting by our assumptions in Equation (10). Therefore, we present a method to offset the negative thresholds to achieve this property. We start by removing the negative threshold parameter, i.e., β = 0. We then repeat the positive and negative representations of the neurons, since TrueNorth neurons are restricted to only one destination axon, and send those outputs back to the same core. The output from the repeated positive representation of a neuron is sent back to its respective negative representation neuron and vice versa to increment the neuron threshold by positive one, thus enabling a neuron potential reset upon *meeting* the negative threshold rather than having to drop below β. This requires two times the original number of neurons in the system and additional axons to accommodate the feedback.

Using this technique, we represent positive and negative outputs via neurons. However, we additionally need inputs that reflect strictly positive or strictly negative values for a recurrent system representing values in this way. We therefore also duplicate our axons to represent both positive and negative values of a variable. This process is similar to having positive and negative representations for neurons: we repeat axons with opposite signs for synaptic weights.

**Figure 4:**
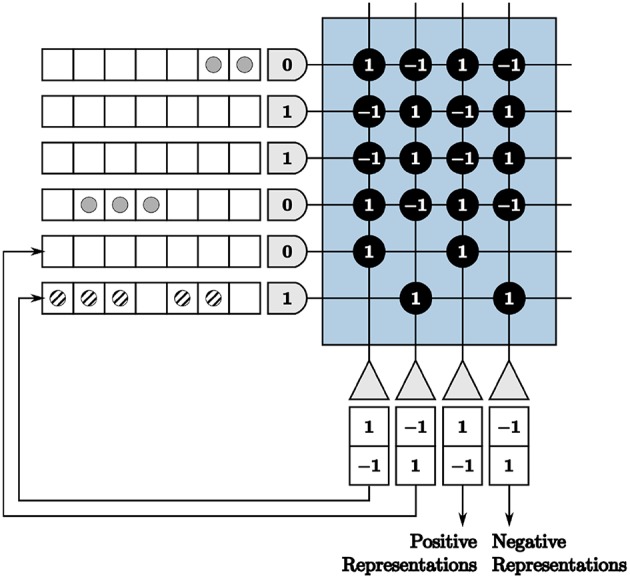
A core with repeated axons and neurons to accommodate positive and negative inputs and outputs.

We show an example of repeated neurons and axons in Figure 4, where the input is [2 –3] multiplied by [1 –1]. Positive inputs are represented by the first two axons and negative inputs by the second two. The first two neurons are fed back to the remaining axons, designated for feedback to create symmetric thresholds. The outputs of these neurons are displayed in the appropriate axon destination tick locations. The last two neurons are used as positive and negative representations of outputs, respectively. This technique results in the correct computation for both positive and negative inputs and outputs.

Each value in our LCA corelet is programmed with the appropriate positive and negative representation of axons and neurons. Each element is also programmed with the appropriate feedback of the neuron outputs to ensure that the positive and negative representations' neuron states are the same every clock cycle. We explain the remainder of this work assuming all incoming and outgoing values are positive to adequately convey programming techniques. However, the reader should note that resources are used on the TrueNorth chip to accommodate sign and feedback requirements.

## 5. LCA Processing Units

Three functional processing units are developed to map the LCA to TrueNorth. The first processing unit performs fixed point integer vector-matrix multiplication (VMM) in what is essentially a collection of binary crossbar arrays. The non-linear soft-threshold processing unit is used to constrain coefficients and compute the sparse approximation of the signal in the LCA. The on-chip memory processing unit enables the implementation of dynamical based iterative algorithms on the TrueNorth.

### 5.1. Vector-Matrix Multiplication With Increased Precision

**Figure 5:**
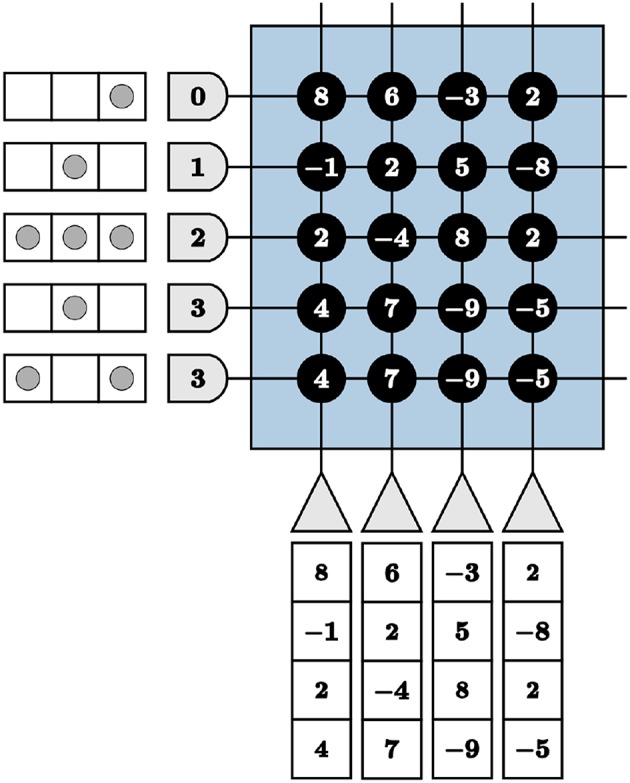
Restricted precision for vector-matrix multiplication if implemented using synaptic weights directly on the TrueNorth chip.

We are faced with programming complexities when mapping iterative linear algebra functionality to TrueNorth. Vital to our LCA corelet for both the initial projection and inhibition functions, adapting the VMM computations to TrueNorth requires novel programming techniques for matrices with columns containing more than four different values. This constraint is due to there being only four synaptic weights per neuron and four axon types to choose from in a TrueNorth core. For example, if we directly use synaptic weights to program a VMM on TrueNorth, we would transpose the matrix by which to multiply and assign synaptic weights the values of the matrix. However, any axons assigned the same type will take on the same synaptic weights if connected to the same neuron. We visualize the VMM

$$M\times v=\left[\begin{array}{ccccc} 8 & -1 & 2 & 4 & 4\\ 6 & 2 & -4 & 7 & 7\\ -3 & 5 & 8 & -9 & -9\\ 2 & -8 & 2 & -5 & -5 \end{array}\right]\;[\begin{array}{c} 1\\ 1\\ 3\\ 1\\ 2 \end{array}]$$

in Figure 5. The last two axons are assigned the same type, therefore the last two columns in the multiplication matrix are identical, limiting the precision of a VMM.

Consequently, we use three layers of cores in series to achieve 9-bits signed precision multiplication matrices. The first layer converts the integer values of the matrix to unsigned binary values. The second layer applies the first set of weights {8 4 2 1}, to the first and second halves of the binary values. The third layer applies the significance of the four most significant and the four least significant bits.

**Figure 6:**
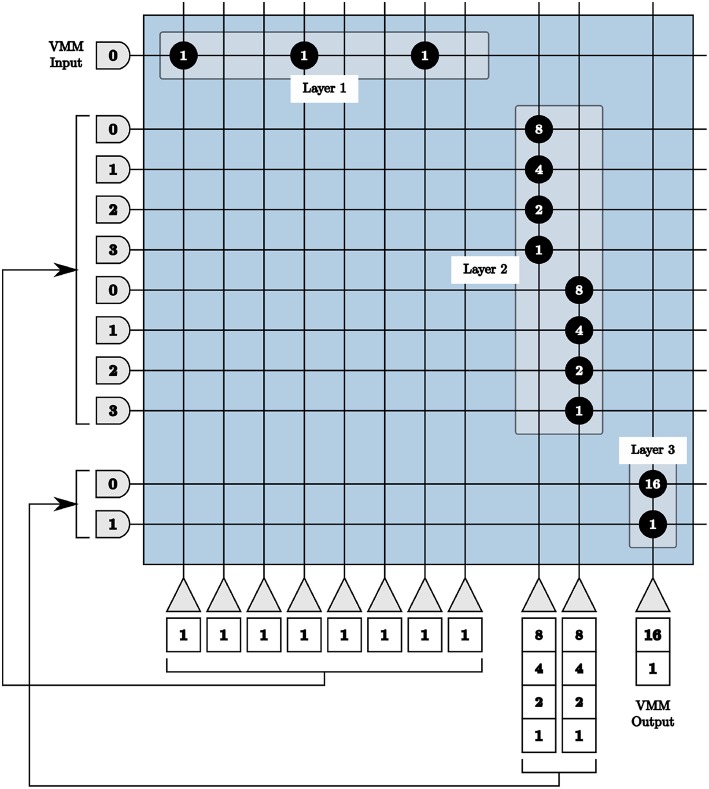
All layers of our vector-matrix multiply overlaid onto one crossbar array, representing one multiplication matrix element with a binary value of 146. Only the positive representations of inputs and outputs are shown.

To program the TrueNorth chip with the binary representation in the first layer, one axon and eight neurons are used. Each neuron represents one bit, where a binary zero signifies no synaptic connection or a binary one denotes a connection between the axon and that neuron with a synaptic weight of one depicted using the value 146 (binary 10010010) as an example shown as *Layer 1* in Figure 6. For perfect precision, in all VMM layers thresholds are α = 1 with linear resets to neuron potentials. If we send an input into the first axon in Figure 6 using these parameters, we will see one output spike each from the first, fourth, and seventh neurons.

The output of the first layer is sent to the axons of the second layer in order. Each set of four axons are connected to one neuron with synaptic weights {8 4 2 1}, shown as *Layer 2* in Figure 6. If we continue our example from above, the first, fourth, and seventh axons of the second layer will receive one input spike, the first neuron in the second layer will update to a potential of 9 using 8 × 1 + 1 × 1, and the second to a potential of 2 using 2 × 1.

The output of the second layer is then sent to the axons of the third layer, with each set of two neurons connecting to the same neuron with weights {16 1}, shown as *Layer 3* in Figure 6. Following the same example, over time the first axon in the third layer will receive nine input spikes from the first neuron of the second layer with a synaptic weight of 16. The second axon of the third layer will receive two input spikes from the second neuron of the second layer with a synaptic weight of one. Both of these axons are connected to the only neuron in the third layer, resulting in a total of 16 × 9 + 1 × 2 = 146 spikes, therefore calculating the original single input spike multiplied by the binary value 146.

In the first layer, eight neurons are needed per column for positive representation neurons, eight per negative representation neurons, and each of those must also be repeated to create symmetric thresholds. For a 9 × 9 matrix, 288 neurons are needed and cannot be accommodated using the available 256 neurons in a single core. As matrices grow larger, the number of required axons also exceeds the 256 available axons within one core. We therefore split our matrix into sub-matrices for each layer and implement each sub-matrix in separate cores in parallel.

The first and second layers are implemented as described above for each sub-matrix independently. The third layer is used to not only apply the significance of the binary values in the matrix, but also to reunite each column of the vector-matrix multiply. The third layer produces positive and negative representations of resultant values for each column of the VMM in parallel. The output spikes from each layer three core are concatenated for the full vector solution.

This method for implementing 9-bits signed VMM is used in our LCA architecture to represent the matrix Φ in the scaled initial projection **b**[*n*] = Φ**y**[*n*] computation and to represent the matrix *G* in the scaled inhibition *Gτ***a**[*n*] calculation.

### 5.2. Non-linear Threshold

The non-linear soft threshold in Equation (8) is essential to compute the sparse approximation of the signal in the LCA. For the purposes of explaining our implementation of the LCA soft threshold on the TrueNorth, we assume unit norm dictionary elements for this section, setting *g*_*k*_ = 1 for all dictionary elements.

The soft threshold parameter λ serves as the trade-off between reconstruction error and sparsity of the sparse approximation of a signal. The scaled soft threshold sets any LCA nodes in |τ**u**| < τλ to zero while otherwise adding or subtracting τλ from −τ**u** or τ**u**, respectively. We add the scaled threshold τλ as a user input to our TrueNorth mapping such that user-determined parameters are not required prior to programming the hardware.

For instances where |τ**u**| exceeds the soft threshold τλ, we can use τ**u** and τλ as inputs to the soft threshold core with synaptic weights of one and negative one respectively. This results in correct computation of the soft threshold with the TrueNorth linear neuron potential resets in Equation (10) by the computation τ**a** = τ**u** − τλ. For example, if τ*u*_*m*_ = 5 and τλ = 4, the resultant sparse approximation for that node *a*_*m*_ = 1 is correct. However, due to the non-linearity of the LCA soft threshold, we cannot simply implement subtraction for cases in which the node state does not exceed the soft threshold. Suppose the values are instead τ*u*_*m*_ = 4 and τλ = 5. The correct sparse approximation for that node would therefore *a*_*m*_ = 0, but would instead be computed on TrueNorth as *a*_*m*_ = −1 if we use the same method as that of the prior case.

As a result, we set positive and negative thresholds to be one and zero respectively, choose a hard reset of zero for neuron potentials in Equation (12), and perfectly align the spikes of τ**u** and τλ. The method by which we perfectly align spikes is as a result of the core outputs we explain in detail in the next section. For the purposes of explaining the soft threshold, however, assume that incoming spikes are either entirely positive or entirely negative as well as sequential (i.e., if the value represented is 10, all 10 spikes occur in 10 sequential TrueNorth ticks). The soft threshold core neurons will never receive an increase in neuron potential greater than one for a single tick, therefore the hard reset upon reaching α = 1 will accurately compute positive results. Any neuron potentials that fall below zero are reset to zero:

(12) $$\begin{array}{l} V_{m}[n+1]=\{\begin{array}{cc} \text{spike},0 & \text{if}\;V_{m}[n]\geq 1\\ 0 & \text{if}\;V_{m}[n]<0\\ V_{m}[n] & \text{if}\;0\geq V_{m}[n]<1. \end{array} \end{array}$$

For unit norm dictionary elements, the above computes the non-linearity correctly. However, for non-unit norm elements, we must consider the values *g*_*k*_ for each element in the LCA soft threshold. To take this into account, one option is to set the prior core's threshold to α = τdiag(*G*) on the neurons that compute τ^2^**u**, resulting in τ**u**/diag(*G*) being one of the inputs to the soft threshold core, while the other input is the user generated τλ/diag(*G*). However, due to the thresholds being greater than one, we now risk the misalignment of spikes due to residual potential between LCA iterations. For instance, if we use thresholds of τdiag(*G*) in the core that computes τ^2^**u**, unless we have an output value that is an exact multiple of τdiag(*G*), we will have neuron potentials remaining between iterations. When this occurs, future iterations' spikes do not align with the user generated input spikes −τλ/diag(*G*) as they now spike at unpredictable times. Therefore, we scale the soft threshold function by τdiag(*G*) and set the thresholds to α = 1 so that the neurons emit spikes sequentially and compute τ^2^**u**, resulting in the soft threshold function $T_{\tau^{2}\lambda }\left(\tau^{2}\text{u}\right)$ implemented in our corelet:

(13) $$\begin{array}{l} \tau^{2}g_{k}a_{k}[n]=\{\begin{array}{cc} \tau^{2}u_{k}[n]-\text{sign}(u_{k}[n])\tau^{2}\lambda & \text{if}|\tau^{2}g_{k}u_{k}[n]|\geq \tau^{2}\lambda \\ 0 & \text{if}|\tau^{2}g_{k}u_{k}[n]|<\tau^{2}\lambda . \end{array} \end{array}$$

Inputs to the core are now τ^2^**u** and a series of τ^2^λ user-generated spikes with synaptic weights of positive one and negative one respectively. The first spikes for each input align in the first tick of a time window of *w* ticks. We choose positive thresholds α = τdiag(*G*) and negative thresholds β = 0 with the hard reset above. Any LCA node states that fall within |τ^2^diag(*G*)**u**| < τ^2^λ set the appropriate values to zero, while active nodes emit τ**a** output spikes.

### 5.3. On-chip Dynamic Memory

**Figure 7:**
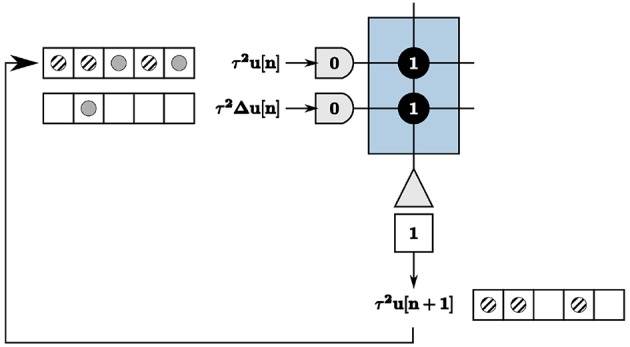
Recurrence resulting in incorrect computations of the node state update.

We address recurrence on the TrueNorth in the context of updating node states of the LCA nodes, calculated by Equation (6). As previously discussed, we wait a time window of length *w* to get exact output values of neurons, computed by counting the number of spikes within each window. This poses complexities with recurrence within the system, illustrated in the example in Figure 7. Inputs τ^2^**u**[*n*] = 2 and τ^2^Δ**u**[*n*] = 1 are denoted by solid gray circles, resulting in outputs τ^2^**u**[*n* + 1] = 3 denoted by diagonally striped circles. The output spikes τ^2^**u**[*n* + 1] are shown as feedback in the appropriate tick locations for the first axon to compute the next iteration's values. The output spikes begin to saturate our time window and cause incorrect calculations within the system for proceeding iterations.

**Figure 8:**
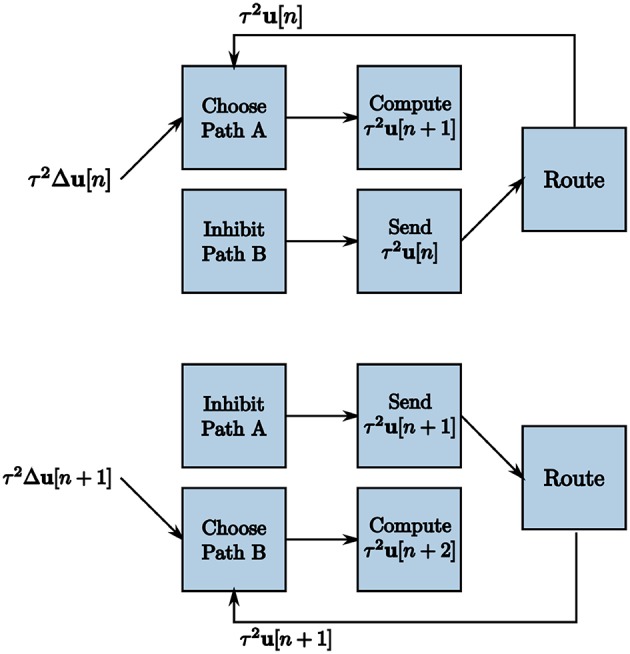
Two subsequent LCA iterations to accommodate recurrence in the system. Triggers enable one path to calculate τ^2^**u**[*n* + 1] over *w* ticks while the other path sends the prior iteration's values τ^2^**u**[*n*] for use in the calculation.

The TrueNorth chip does not have static memory. Instead, each neuron has constantly evolving potentials. Therefore, we must leverage the many neuron parameters to emulate on-chip memory to store and retrieve high-precision values. We create a corelet using a group of cores that computes and stores values τ^2^**u**[*n* + 1] on one path while sending the prior iteration's calculated values τ^2^**u**[*n*] from another in parallel, shown in Figure 8. For one LCA iteration of *w* ticks, one path's neurons are actively spiking while the other path's neurons are inhibited. Suppose Path B is our inhibited path. We send the first core of Path B inhibitory spikes generated by on-chip triggers as input. Inhibitory spikes have a synaptic weight of negative one so that any incoming spikes from τ^2^Δ**u**[***n***] or τ^2^**u**[***n***] are ignored since the threshold of one is not met. Path A does not receive inhibitory input spikes to its first core for this iteration. The incoming spikes are therefore processed and sent to the second core on the same path. The second core of Path A computes τ^2^**u**[*n* + 1] using incoming spikes for *w* ticks. The second core of Path B sends the prior iteration's calculation τ^2^**u**[*n*] forward to the route core, triggered by an incoming excitatory spike.

To ensure that each path is operating correctly at the appropriate times, we create a clock using TrueNorth neurons to generate triggers to send inhibitory and excitatory spikes to each path. To inhibit one path for *w* ticks, *w* inhibitory spikes are required starting at even multiples of *w* and *w* inhibitory spikes are required to occur starting at odd multiples of *w* for the other path. The largest synaptic weight we can choose is positive 255 and our window sizes are most times larger. We choose a leak of one and a positive threshold of α = 255 for a first neuron and send the output spikes to a second neuron with a synaptic weight of one and a positive threshold α = 2*w*/255. The output of the second neuron is therefore one spike every *w* ticks. To emit spikes on odd or even iterations only, we create two clocks with initial potentials of *w*/255 and zero, respectively.

**Figure 9:**
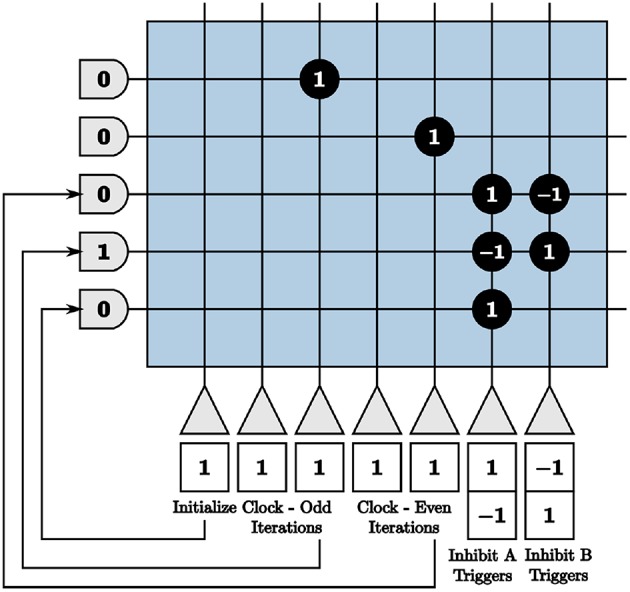
Programmed core that produces inhibition triggers for the first blocks in both paths of the on-chip dynamic memory processing unit.

We use these clocks to generate the on-chip inhibition triggers by sending the output to neurons that constantly emit spikes if turned on and otherwise do not spike, shown in Figure 9. We use initial potentials of negative one and positive thresholds of α = 0 for the inhibition trigger neurons. The inhibition trigger neurons are therefore turned on with an input spike given a synaptic weight of one and off with an input spike with a synaptic weight of negative one from the clock neurons. The initialize neuron is used to begin the alternating inhibition triggers at the appropriate time step for inhibition to occur properly.

The process of computing and sending information from the second core of each path requires a more complex set of on-chip triggers. To compute τ^2^**u**[*n* + 1] in Path A's second core, we want the membrane potential to start at −*w*. All incoming spikes add and subtract within the neurons for *w* ticks without reaching threshold until the next iteration. After *w* ticks, the membrane potential of neuron *m* is $-w+\tau^{2}u_{m}\left[n+1\right]$. To send spikes from Path B's second core in parallel, we send a trigger of user-generated spikes that increases the membrane potential by *w*. In the previous iteration, we compute $\tau^{2}u_{m}\left[n\right]$ in Path B and store the value in the neuron potentials of the second core. With a linear reset and a threshold of one, Path B now sends $-w+\tau^{2}u_{m}\left[n\right]+w=\tau^{2}u_{m}\left[n\right]$ spikes to the route core. The route core sends these spikes back to Path A's first core to compute $\tau^{2}u_{m}\left[n+1\right]$ in parallel.

Once Path B's second core has sent all $\tau^{2}u_{m}\left[n+1\right]$ spikes to the route core, the membrane potential settles to zero until the next iteration. However, we again have both positive and negative representations of the output of the sub-function, therefore our neuron potentials have residual negative potentials for the representation that does not spike given the threshold of one. Suppose the positive representation of a neuron appropriately calculates $\tau^{2}u_{m}\left[n+1\right]$ for the output value. The negative representation of the neuron is left with residual potential of $-\tau^{2}u_{m}\left[n+1\right]$. When we move back to compute mode for the next iteration, we have a starting potential of $-w-\tau^{2}u_{m}\left[n+1\right]$. Switching back to send mode, we see $-w-\tau^{2}u_{m}\left[n+1\right]+\tau^{2}u_{m}\left[n+2\right]+w=-\tau^{2}u_{m}\left[n+1\right]+\tau^{2}u_{m}\left[n+2\right]$ rather than the desired $\tau^{2}u_{m}\left[n+2\right]$. If a change of sign occurs in a node's dynamics, this problem causes the system to converge to an incorrect solution.

To correct the problem, we feedback any output spikes of the positive representation neurons to the negative representation neurons and vice versa with a synaptic weight of one as similar to what we described in section 4.2. During the iteration where the core is sending information, the negative representation neuron potentials from our example will move toward zero by a neuron potential change of positive one for each time step, mirroring the positive representation neuron potentials. This starts the next count iteration correctly at *V*_*m*_ = 0 for both the positive and negative representations.

**Figure 10:**
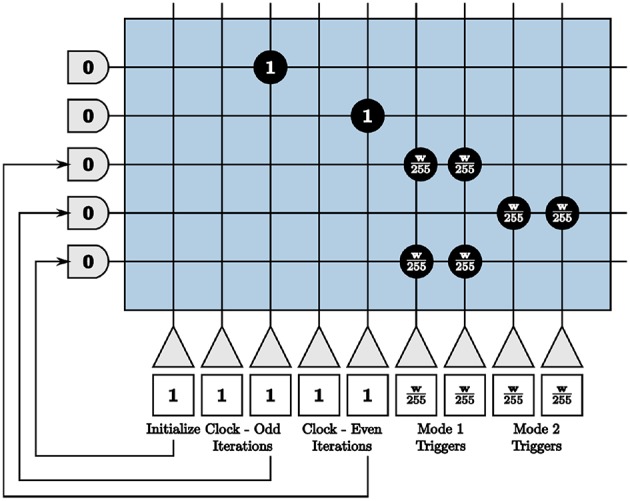
Programmed core that sends send and compute triggers to the second blocks in both paths of the on-chip dynamic memory processing unit.

Because our largest positive and negative synaptic weights are integers +255 and −255 respectively, we set our window size as a multiple of 255 ticks for the entire system. We then send the required number of input spikes to send neuron membrane potentials to −*w* and +*w* for the compute and send modes by the triggers generated in Figure 10. Clock neurons are programmed as before in Figure 9. The different modes represent triggers that activate one path while inhibiting the other and vice versa. The initialize neuron is again used to begin the triggers at the appropriate time step for spikes to align properly.

The on-chip memory processing unit reported is essential to implementing iterative algorithms on the TrueNorth and neuromorphic hardware architectures comparable to the TrueNorth. This processing unit lays the framework for future exploration of computational architectures inspired by the nervous system.

## 6. Results

Using our methods, experimental results of the LCA algorithm mapped to the TrueNorth, which offers limited precision, fixed-point arithmetic, compare favorably with results using floating-point computations on a general purpose computer. We demonstrate the success of the LCA corelet on TrueNorth for dictionaries containing randomly distributed values {–1,0,1} with up to 100 nodes, valuable for use in signal processing applications such as compressed sensing that use image patches as input data. We compare the LCA node dynamics **u** computed by the TrueNorth to the dynamics of a discrete LCA system. We consider our results a success if the LCA node dynamics match, proving that our LCA system on the TrueNorth converges to the same and therefore correct sparse approximation of the original signal.

**Figure 11:**
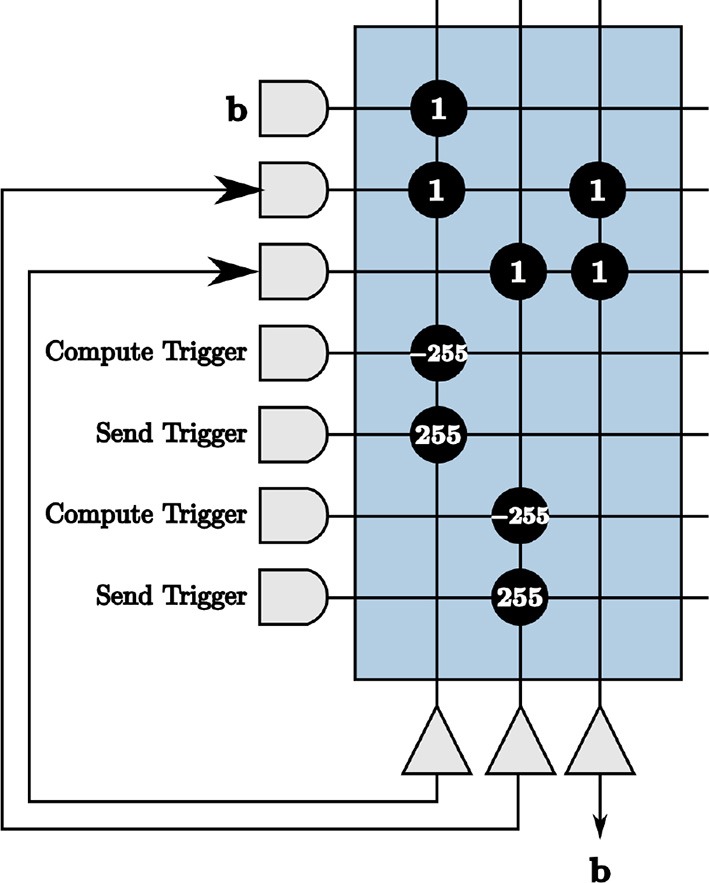
The initial projection repeated on-chip using principles from our on-chip memory corelet.

We choose a constant signal **y** as input to the LCA corelet. The initial projection *b* is therefore also the same, and must be repeated at the beginning of every time window for accurate computation of each LCA iteration. We use the principles from creating on-chip memory in section 5.3 to repeat the initial projection values periodically on the hardware. The user therefore only sends the signal as input for one iteration, our 9-bit signed vector-matrix multiply corelet computes the initial projection once, and we implement a periodic repeater to send the values at the beginning of every time window, shown in Figure 11. For dynamic inputs, we remove the repeater from our corelet.

The soft threshold τ^2^λ is an additional input to the corelet such that the trade-off between reconstruction error and sparsity of the sparse approximation of a signal can be determined at run-time by the user. For this case, the soft threshold remains constant, therefore periodic repetition is used in the same way as for the initial projection. These units are combined to create the final LCA corelet shown in Figure 1.

**Figure 12:**
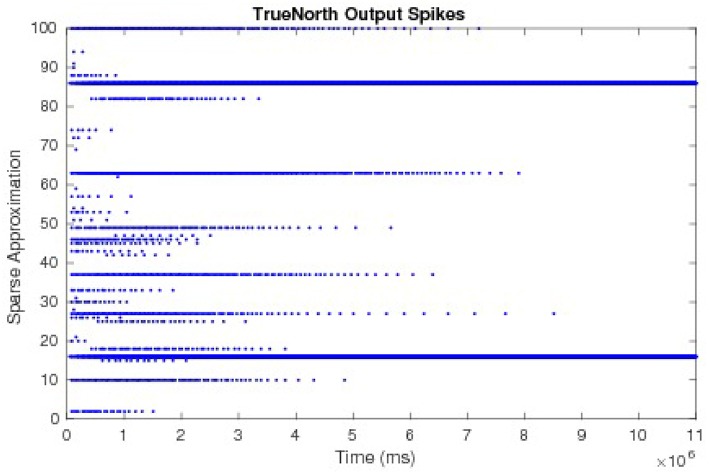
The output spikes from the LCA corelet on the TrueNorth chip, representing positive and negative representations of τ^2^**u**.

To compare and evaluate the node dynamics of each LCA system, we repeat neurons within the sub-function that computes the LCA node dynamics, set thresholds to one, and send output spikes as outputs of the system. We count the number of spikes in each window of *w* ticks to determine the evolving values at each iteration. The resultant node dynamics represented by output spikes from the TrueNorth for an example of a 50-node system is shown in Figure 12. Pins 1 to 50 and pins 51 to 100 are outputs that represent the positive and negative representations of τ^2^**u** respectively. We observe that pins 16 and 86 are the only pins that emit spikes as the system evolves over time, while other pins spike early on but decrease to zero due to inhibition performed by LCA nodes.

**Figure 13:**
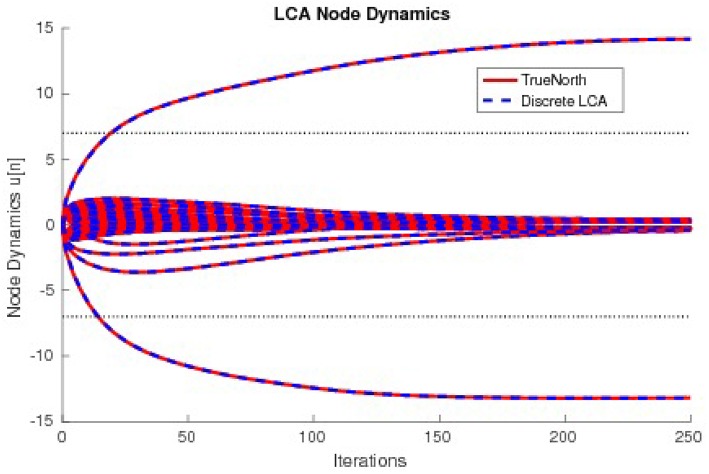
The node dynamics of an LCA system with a 33 × 50 dictionary compared to a discrete LCA system. Input signals are *y* = 14 × Φ_16_−13 × Φ_36_ and parameters are τ = 13 and λ = 7.

The values τ^2^**u**, calculated by counting spikes within each window are divided by τ^2^diag(*G*) and overlaid onto discrete LCA node dynamics computed on a general purpose computer with the same parameters. Node dynamics falling between the dashed lines denote those that fall below the LCA threshold and therefore do not contribute to signal reconstruction. Our system produces identical curves to that of the discrete LCA for hundreds of examples with randomly chosen dictionaries, random input signals generated by a linear combination of one to five dictionary nodes with random weights, and randomly chosen parameters τ and λ, proving that we correctly compute the sparse approximation of a signal for LCA systems. We show the matching node dynamics for a single example in Figure 13, which are the resultant values from the spikes from Figure 12.

### 6.1. Chip Utilization

Each sub-function of the LCA has a limit on the number of LCA nodes it can support if using one core per sub-function. Each core is limited to the aforementioned 256 axons and 256 neurons. By careful indexing, we can expand each sub-function across multiple cores that operate in parallel. For this work we use the original Neurosynaptic System, 1 million neuron evaluation platform (NS1e), limited to 4096 cores. However, multiple NS1e boards can be connected to run networks in parallel or connected in a grid-fashion to execute much larger networks than can be implemented on a single board if more neurons are needed for a particular application. To date, 16 boards have been connected using both methods. The NS1e-16 system connects 16 boards to run many network instances in parallel. The Neurosynaptic System 16 million neuron evaluation (NS16e) consists of three boards with TrueNorth chips in a 4 × 4 grid, offering a platform for networks 16 times larger than one NS1e board (Sawada et al., 2016). Therefore, scaling the Locally Competitive Algorithm as implemented in this work can be done to accommodate much larger dictionaries or larger input signals.

Throughout our corelet, each individual neuron emits spikes and each axon receives spikes representative of the positive or negative representation of an LCA variable for a specific LCA node. However, connections between axons and neurons might not be constrained to the same LCA node. For example, in a vector matrix multiply, we see that a single input is connected to neurons that are representative of several distinct node's LCA variables.

We call axons that connect to neurons that represent the same LCA node as itself *unique axons* and denote the number of such axons per LCA node as Ax_*u*_. We call those axons that connect to more than one LCA node *common axons* and denote the number of such axons as Ax_*c*_. We denote the number of TrueNorth neurons required per LCA node within a sub-function as *N*. The maximum number of LCA nodes we can represent in a single core for a specific sub-function is calculated by

(14) $$\begin{array}{l} \text{Maximum LCA nodes}=\min\left(\frac{256-{\text{Ax}}_{c}}{{\text{Ax}}_{u}},\frac{256}{N}\right). \end{array}$$

We show the number of nodes that can be represented using one core for each sub-function's based on these parameters in Table 1.

**Table 1:** Limitations on the number of LCA nodes a sub-function can accommodate per core.

**Sub-function**	**N**	**Ax_*u*_**	**Ax_*c*_**	**Maximum LCA nodes**
VMM - Initial Projection, Layer 1	32	16	Length(**a**) × 2	8
VMM - Initial Projection, Layer 2	16	8	0	16
VMM - Initial Projection, Layer 3	4	4	0	64
Update τ^2^Δ**u**	6	8	0	32
Update τ^2^**u**, τ**u**, Paths' 1st Cores	4	4	1	63
Update τ^2^**u**, τ**u**, Paths' 2nd Cores	4	6	2	42
Update τ^2^**u**, τ**u**, Route Core	10	2	0	25
Soft Threshold	4	2	1	64
VMM - Inhibition, Layer 1	32	16	Length(**a**) × 2	8
VMM - Inhibition, Layer 2	16	8	0	16
VMM - Inhibition, Layer 3	4	4	0	64

We efficiently program the sub-function's cores by programming the maximum LCA nodes to each sub-function's core. This minimizes the resources required to run the LCA on the TrueNorth chip, leaving additional cores available to perform other processing tasks in parallel. For a dictionary with 66 inputs and 100 LCA nodes (computing the sparse approximation for a signal approximately the size of an image patch), our implementation requires 113 cores, falling far below the available 4096 cores available.

### 6.2. Power Consumption

We measure power for several dictionary sizes and show results in Table 2. The total power is calculated by scaling the leakage power by the number of cores actually used, where *P* is power and *P*_*total*_ = *P*_*active*_ + *P*_*leak*_ * *N*_*cores*_/4096 (Cassidy et al., 2014). This low-power consumption offers our implementation of the LCA on TrueNorth as a feasible choice for embedded systems signal processing applications.

**Table 2:** Power consumption of the LCA implemented on the TrueNorth chip.

	**Average total power (mW)**	
**Dictionary size**	**Operating at 0.8 V**	**Operating at 1 V**
12 × 18	0.343	0.726
33 × 50	0.623	1.326
66 × 100	1.657	3.537

## 7. Discussion

In this work, we present a scalable implementation of the neurally-inspired LCA sparse coding algorithm on a non-von Neumann biologically-inspired architecture, the TrueNorth. The LCA network is recurrent and neurons compete to contribute to the sparse approximation by means of lateral inhibition. While the TrueNorth was developed to successfully deploy neural networks, the LCA architecture differs from that of deep and convolutional neural networks for which the TrueNorth end-to-end ecosystem typically targets, requiring a novel design methodology to map the LCA to the TrueNorth.

As briefly mentioned in the introduction, the LCA has been implemented on FPAAs (Shapero et al., 2012, 2013) to perform sparse reconstruction using dictionaries of up to 12 inputs and 18 LCA nodes. The FPAA design consumes 3.02 mW of power for a 12 × 18 dictionary while our work consumes 0.343 mW for the same sized dictionary (see Table 2). However, for a theoretical FPAA design with a 666 × 1000 dictionary, power consumption is estimated 9.79 mW. Our design uses 1.657 mW for a 66 × 100 dictionary. We can linearly scale the power consumption to do a purely theoretical comparison to the FPAA results, resulting in higher power for the LCA on TrueNorth than for the FPAA design.

The time required to converge to the correct solution is not ideal for applications requiring real-time or near real-time calculations. This is simply a limitation of the TrueNorth architecture for this application. Architectural improvements on the TrueNorth system as well as implementation of the algorithm in other neuromorphic architectures such as the reconfigurable devices discussed in Cassidy et al. (2013a) or BrainDrop Neckar et al. (2019) may enable orders of magnitude improvements in speed over the one millisecond clock the current TrueNorth architecture offers. The clock speed is a consequence of the hardware's inherent speed/power tradeoff and the ability for parallel computations rather than serializing the neuron state updates. Further improvements may also be made by experimenting with different values of τ. In this work the values were taken from Balavoine et al. (2012).

Given the constraints of the TrueNorth hardware and the impact to computation time, one might consider instead utilizing a system with fixed-point arithmetic. However, a recent paper on hardware AI (Sanni and Andreou, 2019) provides insight on the benefits of these neuromorphic architectures. An 8-bit average neuromorphic, charge-based mixed-signal multiplier designed, fabricated, and tested in 16nm FinFET technology outperforms an 8-bit fixed point digital multiplier that is synthesized using standard library cells in the same technology in terms of energy efficiency. This suggests that as the field of neuromorphic engineering matures, implementing neural algorithms much like the LCA on neuromorphic platforms using the design methodologies shown in this paper will create meaningful efficiencies.

Dictionaries with different properties than those used in the LCA system on the TrueNorth are useful for other signal processing applications, therefore this paper provides a useful template for implementing other algorithms on crossbar-based hardware architectures. For example, in some sparse coding literature the dictionary consists of elements that capture common structures and patterns in the data (Olshausen, 2003), much like the localized, oriented, and bandpass receptive fields of the mammalian primary visual cortex (Field, 1994). These fields can be accurately modeled by Gabor-like transforms (Field, 1987). For a dictionary to produce sparse representations that overcome the issues of changes in position, size, and orientation, an overcomplete set of vectors that consist of various dilations and translations of such receptive fields are effective (Simoncelli et al., 1992). These dictionaries take on a wider variety of values that may not be within the {–1,0,1} framework as we use in this work. The initial projection multiplication matrix values will therefore take on larger values than 1 and the values of the inhibition matrix will also grow larger and might even require higher precision than our offered 9-bits signed. Using our methodology, however, more neurons can be utilized to achieve even higher precision if necessary. The number of neurons per LCA node in each layer will increase and therefore the number of cores required to compute the sparse approximation will also increase.

The general techniques to map basic iterative linear algebra and dynamical systems based algorithms within the constraints of the TrueNorth can be used in a variety of other applications as well. Recently, work has been developed to implement the Neural Engineering Framework onto the TrueNorth architecture (Fischl et al., 2018), offering an abstraction for users to perform neural modeling on the hardware. The VMM was utilized as well as other concepts required for this work, such as positive and negative representations of a value, triggers, dynamic memory, and resets as utilized in the MUX component of this work. The VMM functionality is especially useful for alternative applications do to the presented flexible design. For example, if only positive multiplication results are required for a given situation, many neurons and axons can be removed from the design described here. If 4-bit arithmetic is sufficient for an application, the third layer of the presented VMM can be entirely removed. One example that has used this design on the TrueNorth with 4-bit unsigned arithmetic performs Word2vec word similarities (Andreou et al., 2016; Mendat et al., 2018). A 95,000 word dictionary is mapped to one TrueNorth chip using a low-precision variant of the VMM, and words that are similar to an input word are rapidly computed on the TrueNorth.

To summarize, the paper's main focus is the creation of a scalable iterative algorithm (LCA) suitable for crossbar array-based neuromorphic hardware devices. In addition, methodologies are shown to achieve higher precision calculations than those permitted by the inherent limitations of the TrueNorth hardware through encoding and decoding the signal using large time windows. Techniques for implementing non-linear neuron thresholds as well as to accurately map recurrent networks onto the TrueNorth are detailed in this work. As the field of neuromorphic engineering matures, the design methodology detailed in this paper will create energy efficiencies desirable to communities that require similar functionality at low-power.

## Author Contributions

KF independently programmed all computational units with the exception of the vector-matrix multiplication. DM wrote the initial 8-bit unsigned VMM code and KF extended to 9-bit signed VMM, suitable for matrices of any size. KF was supervised by DA and advised by AA, CR, and JR. DM was supervised by AA. All authors jointly did the conceptual work and wrote the paper.

### Conflict of Interest Statement

The authors declare that the research was conducted in the absence of any commercial or financial relationships that could be construed as a potential conflict of interest.
